# 4-(Carboxy­meth­oxy)anilinium bromide

**DOI:** 10.1107/S1600536810018726

**Published:** 2010-06-05

**Authors:** Li Zhang

**Affiliations:** a Ordered Matter Science Research Center, Southeast University, Nanjing 210096, People’s Republic of China

## Abstract

In the title hydro­bromide salt, C_8_H_10_NO_3_^+^·Br^−^, the positive charge resides on the N atom and the carboxyl –CO_2_ end of the cation carries an H atom. In the crystal, N—H⋯Br, O—H⋯Br and N—H⋯O hydrogen bonds link the cations and anions, forming a ladder propagating along the *a* axis.

## Related literature

For background to phase transition materials, see: Jain *et al.* (2008 ▶); Korfer & Fusee *et al.* (1988 ▶).
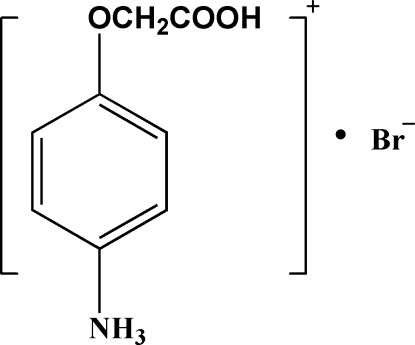


## Experimental

### 

#### Crystal data

C_8_H_10_NO_3_^+^·Br^−^
*M* *_r_* = 248.08
Monoclinic, 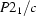

*a* = 6.0182 (12) Å
*b* = 9.6025 (19) Å
*c* = 16.514 (3) Å
β = 94.47 (3)°
*V* = 951.4 (3) Å^3^
*Z* = 4
Mo *K*α radiation
μ = 4.30 mm^−1^
*T* = 293 K
0.40 × 0.30 × 0.20 mm

#### Data collection

Rigaku SCXmini diffractometer
Absorption correction: multi-scan (*CrystalClear*; Rigaku, 2005 ▶) *T*_min_ = 0.5, *T*_max_ = 0.5
9589 measured reflections
2178 independent reflections
1539 reflections with *I* > 2σ(*I*)
*R*_int_ = 0.062

#### Refinement

*R*[*F* ^2^ > 2σ(*F* ^2^)] = 0.039
*wR*(*F* ^2^) = 0.125
*S* = 0.80
2178 reflections
134 parameters
H atoms treated by a mixture of independent and constrained refinement
Δρ_max_ = 0.27 e Å^−3^
Δρ_min_ = −0.85 e Å^−3^

### 

Data collection: *CrystalClear* (Rigaku, 2005 ▶); cell refinement: *CrystalClear*; data reduction: *CrystalClear*; program(s) used to solve structure: *SHELXS97* (Sheldrick, 2008 ▶); program(s) used to refine structure: *SHELXL97* (Sheldrick, 2008 ▶); molecular graphics: *SHELXTL* (Sheldrick, 2008 ▶); software used to prepare material for publication: *PRPKAPPA* (Ferguson, 1999 ▶).

## Supplementary Material

Crystal structure: contains datablocks I, global. DOI: 10.1107/S1600536810018726/ng2777sup1.cif

Structure factors: contains datablocks I. DOI: 10.1107/S1600536810018726/ng2777Isup2.hkl

Additional supplementary materials:  crystallographic information; 3D view; checkCIF report

## Figures and Tables

**Table 1:** Hydrogen-bond geometry (Å, °)

*D*—H⋯*A*	*D*—H	H⋯*A*	*D*⋯*A*	*D*—H⋯*A*
N1—H11⋯Br1	0.95 (4)	2.37 (5)	3.316 (3)	173 (4)
N1—H12⋯Br1^i^	0.99 (5)	2.47 (5)	3.317 (3)	144 (4)
N1—H13⋯O3^ii^	0.91 (4)	1.94 (4)	2.812 (4)	159 (3)
O2—H14⋯Br1^iii^	0.78 (4)	2.46 (4)	3.223 (3)	165 (4)

Symmetry codes: (i) 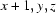; (ii) 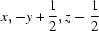; (iii) 
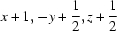
.

**Fig. 1.**
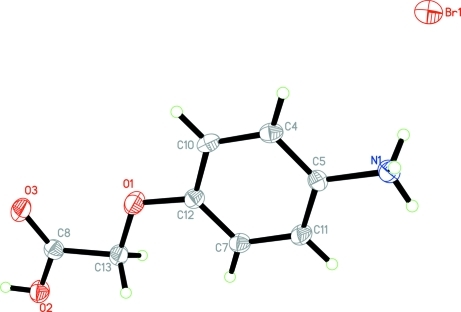
The molecular structure of the title compound, with the atomic numbering scheme. Displacement ellipsoids are drawn at the 30% probability level.

**Fig. 2.**
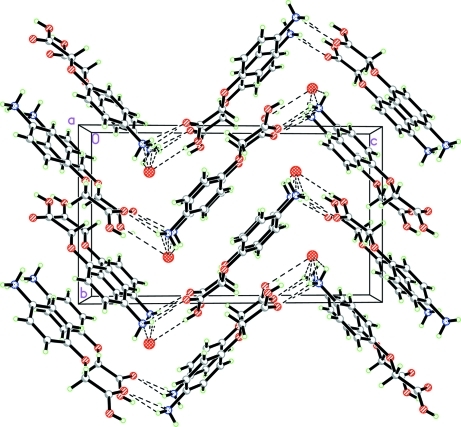
A view of the packing of the title compound, stacking along the a axis. Dashed lines indicate hydrogen bonds.

## supplementary crystallographic information

### Comment

We are interested in the dielectric-ferroelectric materials. Recent studies have revealed that small molecular compounds which have one or more amidogens

probably have this kind of character(Jain *et al.*, 2008 ; Korfer & Fusee *et al.*, 1988). Thus, we want to find aromatic compounds containing amidogens having dielectric-ferroelectric properties. As part of our ongoing studies, we report here the crystal structure of the title compound, The dielectric constant of 2-(4-aminophenoxy) acetic acid bromide compound as a function of temperature indicates that the permittivity is basically temperature-independent, below the melting point (478k-480k) of the compound, the dielectric constant(4.51-8.33) as a function of temperature also goes smoothly, and there is no dielectric anomaly observed, so this compound should be not a real ferroelectrics or there may be no distinct phase transition occurred within the measured temperature range.

The asymmetric unit of the title compound contains one 2-(4-aminophenoxy) acetic acid cation and one bromide anion(fig 1). The non-H atoms of the 2-(4-aminophenoxy) acetic acid are essentially coplanar. In the crystal structure, intermolecular N—H···Br hydrogen bonds link cations and anions to form one-dimensional ladder propagating wavily along the *a* axis direction(fig 2).

### Experimental

ethyl 2-(4-aminophenoxy)acetate (1.95 g) and methanol(30 ml) were added to a round-bottomed flask with a magnetic stirrer bar, then hydrogen chloride(36%) 1.02 g was added into the mixture. The mixture was stirred for 4 h at room temperature. Colourless plate-like crystals of (I) were grown from an ethanol solution of the title compound by slow evaporation at room temperature.

### Refinement

Positional parameters of all the H atoms bonded to C atom were calculated geometrically with C—H = 0.93 to 0.93 \%A, with Uĩso~(H) = 1.2U~eq~(Caromatic,). All other H atom were locatd in a difference Fourier map and refined freely.

### Crystal data

**Table:**

C_8_H_10_NO_3_^+^·Br^−^	*F*(000) = 496
*M**_r_* = 248.08	*D*_x_ = 1.732 Mg m^−^^3^
Monoclinic, *P*2_1_/*c*	Mo *K*α radiation, λ = 0.71073 Å
Hall symbol: -P 2ybc	Cell parameters from 0 reflections
*a* = 6.0182 (12) Å	θ = 3.3–27.5°
*b* = 9.6025 (19) Å	µ = 4.30 mm^−^^1^
*c* = 16.514 (3) Å	*T* = 293 K
β = 94.47 (3)°	Prism, colourless
*V* = 951.4 (3) Å^3^	0.40 × 0.30 × 0.20 mm
*Z* = 4	

### Data collection

**Table:**

Rigaku SCXmini diffractometer	2178 independent reflections
Radiation source: fine-focus sealed tube	1539 reflections with *I* > 2σ(*I*)
graphite	*R*_int_ = 0.062
Detector resolution: 13.6612 pixels mm^-1^	θ_max_ = 27.5°, θ_min_ = 3.3°
ω scans	*h* = −7→7
Absorption correction: multi-scan (*CrystalClear*; Rigaku, 2005)	*k* = −12→12
*T*_min_ = 0.5, *T*_max_ = 0.5	*l* = −21→21
9589 measured reflections	

### Refinement

**Table:**

Refinement on *F*^2^	Primary atom site location: structure-invariant direct methods
Least-squares matrix: full	Secondary atom site location: difference Fourier map
*R*[*F*^2^ > 2σ(*F*^2^)] = 0.039	Hydrogen site location: inferred from neighbouring sites
*wR*(*F*^2^) = 0.125	H atoms treated by a mixture of independent and constrained refinement
*S* = 0.80	*w* = 1/[σ^2^(*F*_o_^2^) + (0.0891*P*)^2^ + 0.5552*P*] where *P* = (*F*_o_^2^ + 2*F*_c_^2^)/3
2178 reflections	(Δ/σ)_max_ = 0.001
134 parameters	Δρ_max_ = 0.27 e Å^−^^3^
0 restraints	Δρ_min_ = −0.85 e Å^−^^3^

### Special details

**Table:**

Geometry. All esds (except the esd in the dihedral angle between two l.s. planes) are estimated using the full covariance matrix. The cell esds are taken into account individually in the estimation of esds in distances, angles and torsion angles; correlations between esds in cell parameters are only used when they are defined by crystal symmetry. An approximate (isotropic) treatment of cell esds is used for estimating esds involving l.s. planes.
Refinement. Refinement of *F*^2^ against ALL reflections. The weighted *R*-factor wR and goodness of fit *S* are based on *F*^2^, conventional *R*-factors *R* are based on *F*, with *F* set to zero for negative *F*^2^. The threshold expression of *F*^2^ > σ(*F*^2^) is used only for calculating *R*-factors(gt) etc. and is not relevant to the choice of reflections for refinement. *R*-factors based on *F*^2^ are statistically about twice as large as those based on *F*, and *R*- factors based on ALL data will be even larger.

### Fractional atomic coordinates and isotropic or equivalent isotropic displacement parameters (Å^2^)

**Table:**

	*x*	*y*	*z*	*U*_iso_*/*U*_eq_	
Br1	0.41951 (5)	0.75195 (3)	0.28076 (2)	0.04135 (18)	
N1	0.9214 (5)	0.6075 (4)	0.29003 (18)	0.0390 (7)	
O1	1.0375 (4)	0.1877 (3)	0.52465 (15)	0.0447 (6)	
C4	0.7915 (5)	0.4697 (3)	0.40446 (19)	0.0329 (7)	
H4A	0.6621	0.5230	0.4021	0.039*	
C5	0.9527 (5)	0.4943 (3)	0.35062 (19)	0.0313 (7)	
O2	1.3878 (4)	−0.0723 (3)	0.61539 (16)	0.0435 (6)	
C7	1.1796 (5)	0.3112 (4)	0.4098 (2)	0.0336 (7)	
H7A	1.3085	0.2575	0.4118	0.040*	
C8	1.2172 (5)	0.0143 (4)	0.6064 (2)	0.0349 (7)	
O3	1.0656 (4)	0.0186 (3)	0.64965 (16)	0.0521 (7)	
C10	0.8249 (5)	0.3660 (3)	0.46127 (19)	0.0344 (7)	
H10A	0.7177	0.3483	0.4976	0.041*	
C11	1.1450 (5)	0.4159 (3)	0.35252 (19)	0.0351 (7)	
H11A	1.2507	0.4332	0.3156	0.042*	
C12	1.0192 (6)	0.2874 (3)	0.4644 (2)	0.0314 (7)	
C13	1.2343 (5)	0.1059 (3)	0.53319 (19)	0.0367 (7)	
H13A	1.3646	0.1654	0.5407	0.044*	
H13B	1.2472	0.0495	0.4851	0.044*	
H11	0.780 (7)	0.650 (5)	0.292 (3)	0.080 (14)*	
H12	1.032 (7)	0.683 (5)	0.299 (3)	0.076 (14)*	
H13	0.931 (6)	0.564 (4)	0.241 (3)	0.063 (13)*	
H14	1.371 (6)	−0.113 (4)	0.656 (2)	0.052 (13)*	

### Atomic displacement parameters (Å^2^)

**Table:**

	*U*^11^	*U*^22^	*U*^33^	*U*^12^	*U*^13^	*U*^23^
Br1	0.0343 (3)	0.0443 (3)	0.0452 (3)	0.00119 (14)	0.00119 (17)	−0.00404 (14)
N1	0.0360 (17)	0.0490 (19)	0.0323 (17)	0.0081 (14)	0.0056 (13)	0.0041 (14)
O1	0.0443 (14)	0.0448 (14)	0.0472 (16)	0.0106 (12)	0.0167 (11)	0.0142 (12)
C4	0.0282 (16)	0.0339 (17)	0.0367 (18)	−0.0005 (14)	0.0039 (13)	−0.0070 (14)
C5	0.0325 (16)	0.0333 (17)	0.0276 (16)	0.0002 (13)	−0.0002 (13)	−0.0009 (13)
O2	0.0461 (15)	0.0446 (15)	0.0407 (15)	0.0091 (12)	0.0089 (11)	0.0073 (12)
C7	0.0310 (17)	0.0349 (17)	0.0354 (18)	0.0039 (14)	0.0065 (13)	−0.0032 (14)
C8	0.0360 (17)	0.0345 (17)	0.0338 (18)	−0.0002 (15)	0.0001 (14)	−0.0032 (14)
O3	0.0454 (14)	0.0727 (19)	0.0403 (15)	0.0104 (14)	0.0163 (12)	0.0135 (13)
C10	0.0267 (15)	0.0396 (18)	0.0380 (18)	−0.0051 (14)	0.0085 (13)	−0.0043 (14)
C11	0.0313 (16)	0.0411 (18)	0.0342 (17)	0.0028 (14)	0.0104 (13)	−0.0009 (14)
C12	0.0320 (16)	0.0307 (15)	0.0317 (18)	−0.0029 (14)	0.0038 (13)	−0.0019 (13)
C13	0.0374 (18)	0.0396 (18)	0.0336 (18)	0.0029 (15)	0.0065 (14)	0.0040 (14)

### Geometric parameters (Å, °)

**Table:**

N1—C5	1.479 (4)	O2—H14	0.78 (4)
N1—H11	0.95 (4)	C7—C12	1.390 (4)
N1—H12	0.99 (5)	C7—C11	1.385 (5)
N1—H13	0.91 (4)	C7—H7A	0.9300
O1—C12	1.379 (4)	C8—O3	1.202 (4)
O1—C13	1.419 (4)	C8—C13	1.505 (4)
C4—C10	1.372 (4)	C10—C12	1.389 (5)
C4—C5	1.386 (4)	C10—H10A	0.9300
C4—H4A	0.9300	C11—H11A	0.9300
C5—C11	1.379 (4)	C13—H13A	0.9700
O2—C8	1.321 (4)	C13—H13B	0.9700
			
C5—N1—H11	111 (3)	O3—C8—C13	124.1 (3)
C5—N1—H12	112 (3)	O2—C8—C13	110.9 (3)
H11—N1—H12	106 (4)	C4—C10—C12	119.9 (3)
C5—N1—H13	105 (3)	C4—C10—H10A	120.1
H11—N1—H13	111 (4)	C12—C10—H10A	120.1
H12—N1—H13	112 (3)	C5—C11—C7	119.5 (3)
C12—O1—C13	118.4 (2)	C5—C11—H11A	120.2
C10—C4—C5	119.3 (3)	C7—C11—H11A	120.2
C10—C4—H4A	120.3	O1—C12—C7	124.2 (3)
C5—C4—H4A	120.3	O1—C12—C10	115.1 (3)
C11—C5—C4	121.3 (3)	C7—C12—C10	120.8 (3)
C11—C5—N1	118.6 (3)	O1—C13—C8	107.1 (3)
C4—C5—N1	120.0 (3)	O1—C13—H13A	110.3
C8—O2—H14	105 (3)	C8—C13—H13A	110.3
C12—C7—C11	119.2 (3)	O1—C13—H13B	110.3
C12—C7—H7A	120.4	C8—C13—H13B	110.3
C11—C7—H7A	120.4	H13A—C13—H13B	108.5
O3—C8—O2	125.0 (3)		
			
C10—C4—C5—C11	0.6 (5)	C11—C7—C12—O1	−179.0 (3)
C10—C4—C5—N1	−178.8 (3)	C11—C7—C12—C10	0.8 (5)
C5—C4—C10—C12	0.2 (5)	C4—C10—C12—O1	178.9 (3)
C4—C5—C11—C7	−0.6 (5)	C4—C10—C12—C7	−0.9 (5)
N1—C5—C11—C7	178.7 (3)	C12—O1—C13—C8	175.9 (3)
C12—C7—C11—C5	0.0 (5)	O3—C8—C13—O1	−3.0 (5)
C13—O1—C12—C7	1.6 (5)	O2—C8—C13—O1	175.7 (3)
C13—O1—C12—C10	−178.2 (3)		

### Hydrogen-bond geometry (Å, °)

**Table:**

*D*—H···*A*	*D*—H	H···*A*	*D*···*A*	*D*—H···*A*
N1—H11···Br1	0.95 (4)	2.37 (5)	3.316 (3)	173 (4)
N1—H12···Br1^i^	0.99 (5)	2.47 (5)	3.317 (3)	144 (4)
N1—H13···O3^ii^	0.91 (4)	1.94 (4)	2.812 (4)	159 (3)
O2—H14···Br1^iii^	0.78 (4)	2.46 (4)	3.223 (3)	165 (4)

Symmetry codes: (i) *x*+1, *y*, *z*; (ii) *x*, −*y*+1/2, *z*−1/2; (iii) *x*+1, −*y*+1/2, *z*+1/2.
